# Treatment of intrabony defects with modified perforated membranes in aggressive periodontitis: subtraction radiography outcomes, prognostic variables, and patient morbidity

**DOI:** 10.1007/s00784-018-2712-7

**Published:** 2018-10-30

**Authors:** Bartłomiej Górski, Stanisław Jalowski, Renata Górska, Maciej Zaremba

**Affiliations:** 1 Department of Periodontology and Oral Mucosa Diseases, Medical Unversity of Warsaw, Miodowa St 18, 00-246 Warsaw, Poland; 2 Department of Dental and Maxillofacial Radiology, Medical University of Warsaw, Nowogrodzka St 59, 02-006 Warsaw, Poland

**Keywords:** Aggressive periodontitis, Digital subtraction radiography, Intrabony defect, Periodontal regeneration

## Abstract

**Objectives:**

The main objectives of this study were (1) to evaluate bone/graft density alterations by digital subtraction radiography; (2) to determine factors associated with favorable clinical and radiographic outcomes, and (3) to report on patient morbidity after guided tissue regeneration (GTR) in aggressive periodontitis (AgP) patients.

**Materials and methods:**

Adapting a split-mouth design, 30 comparative intrabony defects in 15 patients were randomly treated with xenogenic graft plus modified perforated membranes (MPM, tests) or xenogenic graft plus standard collagen membranes (CM, controls). The time period of observation was 12 months.

**Results:**

There were significant improvements in clinical and radiographic parameters within each group, without intergroup differences. However, higher PPD reduction for three-wall defects was noted in MPM sites (5.22 versus 3.62 mm; *p* = 0.033). Moreover, a significant gain in bone/graft density of 4.9% from 6 to 12 months post-operatively was observed in test sites. Multivariate analysis demonstrated that morphology of intrabony defects was a predictor of CAL gain (*p* = 0.06), while independent prognostic variables effecting changes in bone/graft density were radiographic defect depth (*p* = 0.025) and radiographic angle (*p* = 0.033). The majority of patients reported some discomfort, pain, and edema with mild intensity without any significant differences between treatment modalities.

**Conclusions:**

This study demonstrated enhanced bone/graft density gain after GTR with MPM, which may indicate greater area of new bone formation. Independent variables effecting treatment outcomes were intrabony defect morphology, radiographic defect depth, and radiographic angle.

**Clinical relevance:**

This study supports the regenerative treatment of intrabony defects in AgP patients and identifies some variables with prognostic value.

## Introduction

The main features of aggressive periodontitis (AgP) are rapid rate of disease progression, a discrepancy between the amount of local factors versus periodontal destruction, absence of any systemic involvement and familial aggregation [1]. Owing to its less frequent prevalence, which varies from 0.5 to 2.5%, only few studies have assessed various treatment modalities for this condition [2]. It should be mentioned though, that the 2017 World Workshop on the Classification of Periodontal and Peri-Implant Diseases and Conditions reached the conclusion that there is no evidence to consider AgP as pathophysiologically distinct disease; hence, a case definition of periodontitis should be based on a matrix of periodontitis stage and periodontits grade [3]. As this study was performed long before the new classification, we adhered to previously used system articulated during the 1999 International Workshop on Classification of Periodontal Diseases, which differentiated between AgP and chronic periodontitis (ChP) [4].

Taking into account limited self-healing capability of periodontal intrabony defects, the treatment of choice is guided tissue regeneration (GTR), which aims at restoring lost periodontal tissue. In this procedure, a biocompatible barrier membrane is placed between a surgical flap and root surface to conduct cell recruitment in a selective manner. Up to now, it has been widely accepted that epithelial apical migration and soft tissue cell ingrowth interfere with regeneration [5]. However, recent studies emphasized that regeneration of intrabony defects relies on the interplay of numerous cell lineages derived from various tissue origins. Consequently, the importance of periosteum and gingiva, as a notable source of mesenchymal stem cells (MSCs), which can efficiently contribute to the repair of bone defects, has been highlighted. Periosteum-derived stem/progenitor cells (PDPCs) were found to express *Leptin Receptor*, *Gremlin 1*, and KDR (aka Flk1 or VEGFR2) [6]. Therefore, in course of injury or inflammation, PDPCs, controlled by periostin, would become activated to simultaneously trigger both angiogenic and bone repair processes of periodontal defects [7]. On the other hand, gingival mesenchymal stem/progenitor cells (GMSCs) demonstrated osteogenic potential in the presence of inflammation [8]. In addition to their great efficiency in bone reconstruction and repair for clinical use [9, 10], GMSCs participated in recruitment of bone progenitor cells and other endogenous MSCs, showing unique immunomodulatory and anti-inflammatory functions [11]. However, barrier placement in GTR procedures excludes any contribution of MSCs and biologic mediators from the periosteum and gingival connective tissue, which might affect the favorable outcomes of regenerative procedures in intrabony defects.

Despite growing understanding of periodontal wound healing processes, the results of GTR are associated with a relatively high degree of variability [12]. As a matter of fact, significant individual differences regarding response to the treatment were described in AgP patients [13]. Thereupon, continuous research is being conducted to exploit newer strategies with a view to improve existing treatment concepts. With that in mind, Gamal and Iacono [14] were able to validate the superior clinical performance of novel modified perforated collagen membranes (MPM) in regenerative treatment of intrabony defects in patients diagnosed with severe ChP. Very recently, the authors of the present study demonstrated enhanced defect fill when using MPM compared with standard collagen membranes (CMs) in AgP patients [15]. Moreover, MPM resulted in improved periodontal regeneration in dehiscence defects in dogs with the formation of significantly denser bone trabeculations, more rapid bone maturation, and higher bone surface area, as compared with CM [16]. The overall idea behind this concept was for membrane perforations to allow GMSCs, PDPCs, and growth and differentiation factors to participate in supracrestal regeneration of intrabony defects on the one hand, while to provide greater clot stability on the other hand, all of which are critical factors in promoting periodontal regeneration, which could potentially impinge on the clinical/radiographic features.

The evaluation of GTR efficacy concentrated on changes in clinical parameters, such as clinical attachment level (CAL) gain and probing pocket depth (PPD) reduction, or radiographic outcomes that included radiographic defect depth (DD) reduction and bone fill. Recently, more precise methods of radiographic image analysis have been introduced which depend on digital image subtraction and densitometry. Digital subtraction radiography (DSR) aims for diagnostic accuracy to detect minimal changes in bone density in the area of immediate interest. In this approach to minimize technical errors caused by geometric discrepancy, two highly standardized radiographs are taken at various times and subtracted from one another [17]. The differences in gray shade values might be viewed as changes in bone density. The radiographic subtraction technique detects bone alterations of 5%, with over 90% of sensitivity and specificity, but when bone graft materials with slow resorption rate are used in GTR, DSR may not represent a true bone formation and maturation [18, 19]. On the other hand, it is also vital to take patient-centered outcomes into account when evaluating the effectiveness of GTR. However, so far patient-reported outcomes have been generally overlooked in clinical studies and no randomized clinical trial (RCT) mentioned patient morbidity and perceptions on GTR in AgP.

From a clinician’s point of view, it is of paramount importance to identify and control the variables that might be associated with the outcomes of GTR in order to improve predictability of the treatment. Clinicians need to be acquainted with factors implicated with periodontal regeneration so as to by proper case selection, they may obtain the best clinical results. Manifold variables influence the predictability of treatment outcomes after periodontal regenerative procedures. The main causes of clinical variability are the patient- (plaque control, percentage of bleeding on probing, smoking habit, diabetes mellitus, compliance with supportive periodontal therapy), defect- (tooth type and position, defect morphology, defect depth and width, radiographic angle), and surgery-associated factors. However, the knowledge of variables that may help to predict GTR results in AgP is scarce. As a matter of fact, consensus reports [20] and meta-analysis [21] did not distinguish between regenerative treatment of intrabony defects in patients diagnosed as having AgP or ChP. To the best of the authors’ knowledge, no study has reported so far on the prognostic factors of GTR outcomes in patients with AgP. By the same token, it appears justifiable to determine whether the abovementioned factors yield a similar impact on patients with AgP after periodontal regenerative procedures.

This study is a 12-month RCT of two periodontal treatment modalities of intrabony defects: GTR with MPM (tests) and GTR with CM (controls) in AgP patients. Its clinical and radiographic outcomes have previously been published [15]. The aim of this report was to measure changes in bone/graft density by means of DSR after GTR. We hypothesized that using MPM in GTR may have positive impact on bone/graft density gain. Although this trial was not planned to evaluate prognostic factors contributing to healing of intrabony defects, the paucity of those data makes such analysis well-founded. Therefore, another aim was to identify independent prognostic variables associated with favorable clinical and radiographic 12-month outcomes in AgP. This paper also reports on the post-operative morbidity and patient-centered outcomes.

## Materials and methods

### Study design

This study was designed as a randomized, controlled, double-masked and split-mouth trial and was performed in the Department of Periodontology of Medical University of Warsaw, after receiving the approval by institutional review board (KB/135/2014). The research was carried out in accordance with the Helsinki Declaration of 1975, as revised in Tokyo in 2004. Fifty-two patients referred by general dentists were assessed for eligibility, among which 37 did not meet inclusion criteria, 5 declined to participate, and 2 were excluded for other reasons (long-distance place of residency, work scheduling that made regular appointments impossible). Fifteen subjects who met inclusion criteria were allocated for initial treatment. Patients were informed of the nature, potential risks, and benefits of their participation in the study. All participants signed consent forms. Six weeks after non-surgical therapy, a reevaluation for eligibility was carried out. Adapting a split-mouth design, two comparative intrabony defects from each patient were selected and subsequently treated by one of the two periodontal regenerative treatment modalities in a 2-week time span. One defect was randomly treated with xenogenic graft plus modified perforated membrane (test), and the second defect was treated with xenogenic graft and standard collagen membrane (control) [15]. Randomization was performed before surgical treatment by the statistician, who used a computerized random number generator. Allocation to treatment strategy was sealed in numbered envelopes and disclosed to the periodontist immediately before the surgery. No information on treatment allocation was revealed to the patient. After completion of active treatment, all patients were enrolled in a 12-month maintenance phase, which was provided by a specialist.

Additional information regarding patient flow, sample size calculation, and calibration has been thoroughly described in previous publication in *Clinical Oral Investigations* [15].

### Presurgical procedures

Initial periodontal therapy covered supragingival scaling with personalized oral hygiene instructions until good control of bacterial biofilm was accomplished. Subsequently, scaling and root debridement under local anesthesia with occlusal adjustment, when required, were administrated. Additionally, to all patients, systemic antibiotics were prescribed (amoxicillin 500 mg + metronidazole 250 mg three times daily for 1 week). Six weeks after the completion of non-surgical treatment, a reevaluation was carried out and patients, who fulfilled the study’s criteria, were scheduled for surgical therapy.

### Patient and defect eligibility

Subjects were enrolled in the study if positive for each of the following inclusion criteria: (1) diagnosis of AgP according to definitions of American Academy of Periodontology of 1999 [1]; (2) no systemic diseases; (3) no use of medications affecting periodontal status; (4) no-smokers or smoking < 10 cigarettes/day; (5) not pregnant or lactating; (6) history of periodontitis in parents or siblings; (7) presence of at least two teeth with PPD ≥ 6 mm, CAL ≥ 5 mm, and DD ≥ 3 mm as detected in periapical radiographs; (8) full-mouth plaque score (FMPS) ≤ 20%; (9) full-mouth bleeding score (FMBS) ≤ 20%; (10) tooth had to be vital or properly treated; (11) no furcation involvement; (12) the width of keratinized tissue on the vestibular site of the tooth ≥ 2 mm.

### Clinical recordings

Clinical measurements were taken by the same experienced and calibrated examiner (MZ). Briefly, full-mouth plaque score (FMPS) and full-mouth bleeding on probing score (FMBS) were recorded before the surgery, at 1, 3, 6, and 12 months post-operatively. FMPS was calculated as the percentage of tooth surfaces that exhibited plaque [22]. Likewise, FMBS was evaluated as the percentage of periodontal pockets that bled from the bottom 15 s after careful probing [23]. Clinical parameters were registered at six aspects of each tooth (i.e., distobuccal, buccal, mesiobuccal, distolingual, lingual, mesiolingual) with a graded periodontal probe (UNC probe 15 mm, Hu-Friedy, Chicago. Illinois, USA) and rounded off to the nearest millimeter: (1) PPD (distance from the gingival margin to the base of periodontal pocket); (2) CAL (distance from the cemento-enamel junction to the base of periodontal pocket; (2) gingival recession (GR, distance from the cemento-enamel junction to the gingival margin). Measurements of PPD, CAL, and GR were done at baseline and 12 months after treatment.

Upon completion of intrasurgical debridement, the consecutive measurements were recorded: (1) depth of the defect (distance from the most coronal point of the bony walls surrounding the defect to the deepest point in the defect); (2) width of the defect (distance from the most coronal point of the bony walls surrounding the defect to the root surface); (3) the number of the remaining bone walls of the defect (defects were classified as one-wall, two-wall, and three-wall defects).

### Radiographic measurements

Standardized reproducible digital periapical radiographs were collected from each site with phosphor plates (KaVo Scan eXam, KaVo, Biberach, Germany) with modified film holders and paralleling technique using an x-ray unit operating at 70 kV, 4 mA, and 0.1-s exposure time, prior to surgery and at 6 and 12 months post-operatively. The images were 512 × 460 pixels and had a 256 Gy scale. To index the dentition, a registration material was placed on bite blocks. The radiographs were analyzed using Planmeca Romexis Viewer software (Planmeca, Helsinki, Finland) by an experienced and calibrated clinician (SJ). Anatomical landmarks, which included cemento-enamel junction (CEJ), alveolar crest (AC), and base of the defect (BD), were selected on the radiographs as described by Schei et al. [24]. Two auxiliary lines were drawn, first in tooth axis (AUX1) and second line (AUX2) from AC, perpendicular to AUX1. DD was measured as the distance from the spot where AUX2 crossed the CEJ-BD line to the base of the defect. The radiographic defect angle was calculated between the intersection of CEJ-BD line of the tooth and the delimitation of the wall of the defect [25].

**Fig. 1:**
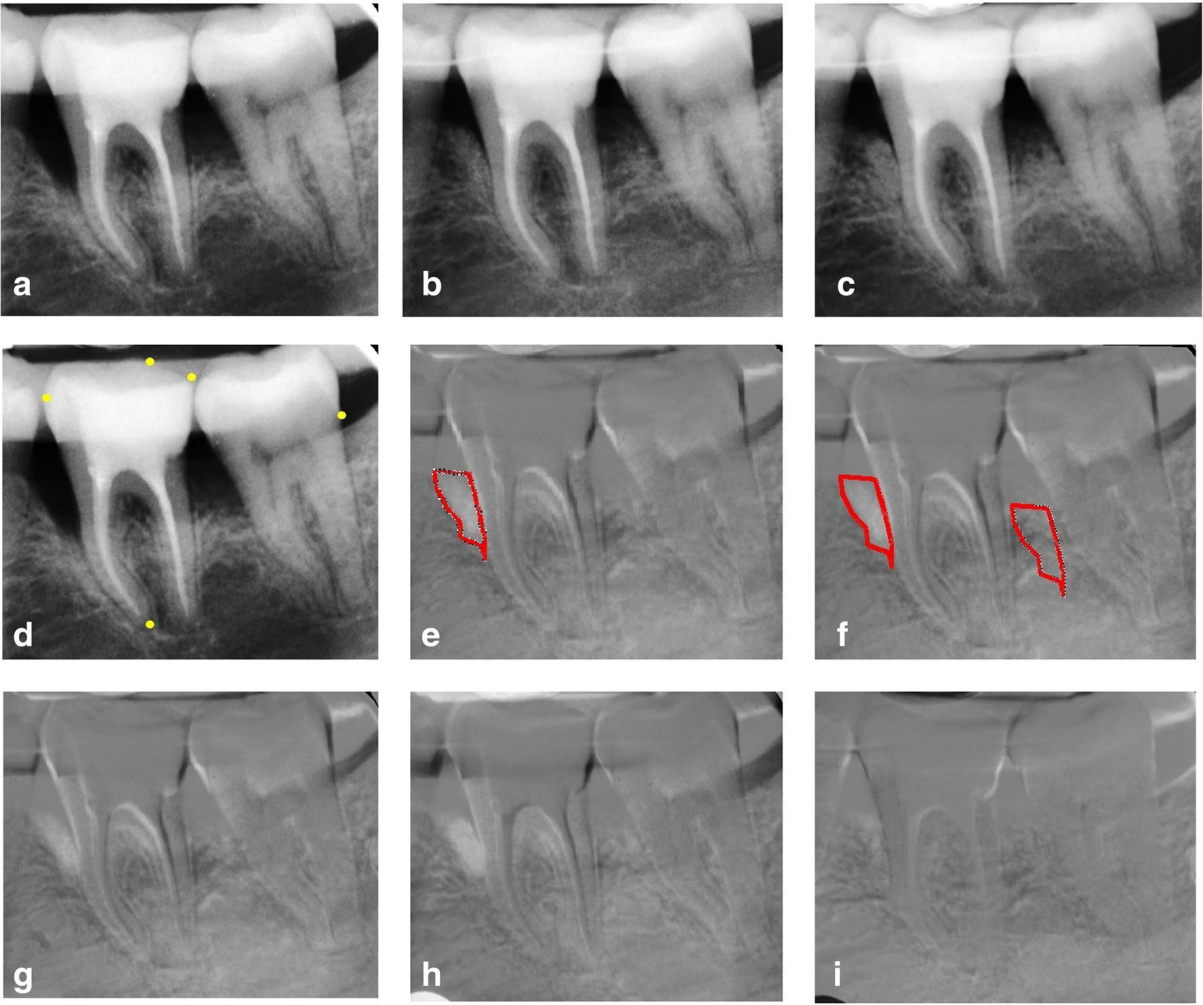
Subtraction radiography of test site (MPM-treated). Intrabony defect on mesial surface of tooth 36. **a**–**d** Radiographs after normalization. **a** Baseline radiograph. **b** Six-month radiograph. **c** Twelve-month radiograph. **d** Fixed points were marked (5 points) on baseline radiograph. **e**–**i** Subtraction images with a brighter area in the region of interest (ROI) representing the region with gain in density. **e** ROI was outlined by an irregular line corresponding to the borders of intrabony defect. **f** The reference ROI of the same shape was drawn on the healthy interradicular space as the region of control. **g** Subtraction of 6-month radiograph from baseline radiograph. **h** Subtraction of 12-month radiograph from baseline radiograph. **i** Subtraction of 12-month radiograph from 6-month radiograph

In ImageJ® (Research Services Branch, NIH, Bethesda, Maryland, USA), automatic normalization correction was performed to optimize brightness and contrast variations for all radiographs taken. With Regeemy-Image Registration and Mosaicking- 0.2.43 software (DP-INPE Sao Jose dos Campos, Brazil and Vision Lab Electrical and Computer Engineering Department, University of California, Santa Barbara, CA), geometric reconstruction was proceeded to compensate for any distortion of the projection with manual selection of five reference points in areas of highly contrasted contours such as the root apices, the cusp tips, and tooth contact points. Radiographs obtained at 6 and 12 months were subtracted from the radiographs taken at the baseline. Moreover, radiographs taken at 12 months were subtracted from the radiographs taken at 6 months. Subsequently, the images were uploaded to ImageJ® software, the region of interest (ROI) was outlined by drawing an irregular line corresponding to the delimitation of the walls of the intrabony defect, and mean gray values were calculated. The operating ROIs were not superimposed on any portion of the tooth surface. The measured area varied from patient to patient, but was the same in one patient site over time (Fig. 1). Afterwards, the reference ROI of the same shape was superimposed on the healthy interradicular bone, which had allegedly not changed during the study and the mean gray value was measured and set up as a reference. The abovementioned value was used to calculate the changes in bone/graft density in the subtracted image. The areas that showed gain in radiographic density (lightened areas) were measured in mm^2^. The percentage of areas with gain in radiographic density detected in post-operative x-rays in relation to baseline defects was calculated.

### Surgical interventions

All defects were treated by one surgeon (BG) in the same manner in accordance with guidelines of minimally invasive surgical technique [26]. Briefly, after the administration of local anesthesia with 4% articaine hydrochloride with adrenaline (1:100000) (Ubistesin Forte 1.7 ml, 3 M ESPE, Saint Paul, Minnesota, USA), the defect-associated interdental papilla was accessed with the simplified papilla preservation flap in narrow interdental spaces (the width of interdental space 2 mm or less) or with the modified papilla preservation technique in large interdental spaces (the width of interdental space > 2 mm). The buccal crevicular incisions were minimally extended and the full-thickness buccal flap was raised. Subsequently, the interdental papilla was elevated with lingual flap. Mucoperiosteal flaps on the facial and lingual aspects were raised to expose 2–3 mm of the alveolar bone beyond the defect margins. Vertical incisions were used only if necessary. Intrabony defects were debrided, followed by meticulous root planing using mechanical and hand instrumentation. Subsequently, deproteinized bovine bone mineral (DBBM, Bio-Oss®, Geistlich Biomaterials, Princeton, New Jersey, USA) were placed to fill the intrabony defects. At this point, an opaque envelope with randomized treatment assignment was opened and the treatment modality was delegated to either covering the defect with trimmed modified perforated collagen membrane (MPM/test, Bio-Gide®, Geistlich Biomaterials) or standard collagen membrane (CM/control, Bio-Gide®). Randomization was accomplished in advance of surgery by a computer-generated table. Membrane perforations in test group were prepared by using a custom-made acrylic template, leaving a coronal occlusive rim of ~ 3 mm [14]. Perforations were made with a standard hand-spreader number 40 (Poldent, Warsaw, Poland). Membrane was adapted in place to cover the defect and 2–3 mm of remaining bone without suturing. After membrane placement, the periosteal incision was made at the base of the buccal flap to advance flap coronally without tension. Primary soft tissue closure was achieved by single modified internal mattress sutures (5/0 polypropylene monofilament suture, Prolene 5/0 16 mm 3/8, Ethicon, Somerville, New Jersey, USA) in the inter-proximal areas and simple passing sutures in vertical incisions.

### Post-surgical care

The patients were provided with meticulous written post-operative instructions to avoid brushing, flossing, and chewing in the treated area. After the surgery, patients received 600 mg ibuprofen and were asked to take a second dose 8 h later. Subjects were requested to rinse with 0.2% chlorhexidine digluconate mouth rinse three times per day for 3 weeks. Sutures were removed after 2 weeks, and patients were instructed to gentle brushing with a soft toothbrush. During the maintenance phase, subjects were monitored once every 2 weeks for 3 months and every 3 months for 1 year. Each session consisted of reinforcement of oral hygiene instructions and supragingival plaque removal.

### Evaluation of post-operative morbidity

Patient perception of post-operative morbidity and satisfaction with treatment were evaluated with a questionnaire administrated 2 weeks after surgery (at suture removal). Respondents were provided with seven single-item visual analogue scales (VAS) that were used to measure the intensity of discomfort, pain, edema, eating, and speech impairment, interferences with daily activities and work, which they experienced during post-operative period [27]. Each VAS consisted of a horizontal line, 10 cm (100 mm) in length, with a statement at each end representing one extreme of the variable being evaluated (e.g., for pain intensity, the scale was anchored by “no pain” as score 0 and “worst imaginable pain” as score 100). The questionnaires were self-completed by the patients who marked a line perpendicular to the VAS line at the point that represented intensity of their experiences. Moreover, patients were asked to write the number of analgesics taken in addition to the first two compulsory tablets. To evaluate patient’s long-term treatment satisfaction, two questions were formulated: (1) “Considering everything, how satisfied are you now with the results of the surgery?”, (2) “If you had to make the decision again, how likely would you be to have this surgery?” [28]. Patients were asked to answer both questions 1 year after surgery.

### Statistical analysis

Statistical analysis was carried out using Statistica 13 [Dell Inc. (2016). Dell Statistica (data analysis software system), version 13 software.dell.com. Any *p* values of less than 0.05 (*p* < 0.05) was considered statistically significant. Normality of distribution was assessed using Shapiro-Wilk test and by visual inspection of histograms. For quantitative variables with normal distribution, mean ± standard deviation (95% confidence interval) was given. For the nominal data, values were provided as the frequency.

The primary outcome variable was CAL gain at 12 months, and the secondary variables were PPD reduction and subtraction radiographic outcomes at 12 months. For statistical analysis, the measurements at the site with the greatest presurgical CAL value were used. By deducting the 12-month values from the baseline values, the 12-month changes in clinical and radiographic outcomes were calculated. Likewise, a positive 12-month change implied a reduction in PPD, a gain in CAL, a decrease in GR, and a reduction in DD. The changes in bone density were evaluated by DSR. Comparisons between MPM-treated and CM-treated sites at the same time points were performed using *t* test for independent variables, while comparisons of changes in time within the same group were evaluated by *t* test for paired data.

A subgroup analysis on defect fill potential depending on defect type was carried out. Due to the number of one-wall and two-wall defects being inadequate for intergroup comparisons, defects were classified as type A (one-wall and two-wall) and type B (three-wall). The analysis was performed with *t* test for independent samples.

To verify the effect of potential predictors on 12-month PPD reduction (≤ 4 versus >4 mm), 12-month CAL gain (> 3 versus ≤ 3 mm), 12-month subtraction radiography outcomes (≤ 70% versus > 70% gain in bone/graft density), and change in bone/graft density in subtraction radiography from 6 to 12 months post-operatively (≤ 0% versus > 0%) multiple logistic regression was applied. The results were presented as odds ratios (ORs) and confidence intervals (CIs) for OR. The analysis was performed for all the treated defects combined, due to their limited number. The model contained clinical and radiographic variables of feasible importance, namely tooth type and tooth position, baseline PPD, CAL and GR, radiographic DD and radiographic angle, morphology of intrabony defects (number of walls), depth and width of intrabony defects, and pattern of early healing (primary/secondary). All of the abovementioned variables were evaluated individually and with multiple regression model. Final regression models were obtained using stepwise selection of predictors with backward elimination. The strict entry criteria excluded the recruitment of patients with inadequate oral hygiene and high residual infection; hence, patient-related factors were not considered in the analysis.

The analysis of patient perception of post-operative morbidity after treatment with MPM or CM was carried out with *t* test for independent samples.

## Results

Fifteen patients (10 women and 5 men, aged 22–49; mean age 37.9 ± 7.95 years) with 30 defects were treated with both MPM and CM GTR surgery in 2 weeks’ time span. All subjects completed the 6- and 12-month follow up. At baseline, FMPS was 8.4% (± 7.6), while FMBS 12.6% (± 7.3) meaning good oral hygiene and low levels of residual infection. No intergroup difference was noted in clinical and radiographic variables at baseline, as well as in defect morphology assessed intrasurgically (Table 1). Healing was uneventful in all subjects. However, membrane exposure was noted at 2 to 3 weeks after surgery in five sites (3 MPM sites and 2-CM sites). Membrane exposures took place in five distinct patients. Exposed areas were treated with 0.2% chlorhexidine solution at the follow-up visits and with daily application of 1% chlorhexidine gel by patients until complete re-epithelialization. From baseline to 12-month follow-up, while the GR showed an increase, PPD, CAL, and DD decreased significantly, but no difference was detected between groups (Table 1). The meticulous description of the abovementioned outcomes were accounted in a separate article published in *Clinical Oral Investigations*. Oral hygiene was maintained within acceptable levels (FMPS < 20%, FMBS <2 0%) throughout the study period (Table 2).

**Table 1:** Clinical, radiographic, and surgical recordings for test and control groups

Variables	Test (*n* = 15)			Control (*n* = 15)			*p* value
	Baseline	12 months	Change	Baseline	12 months	Change	
Clinical measurements^a^							
PPD (mm)	7.4	3.4*	4.0	7.2	3.7*	3.5	0.468
	[6.5–8.3] ± 1.5	[2.8–4.0] ± 1.1	[2.8–5.2] ± 2.1	[6.5–7.9] ± 1.3	[3.2–4.2] ± 0.9	[2.8–4.2] ± 1.2	
CAL (mm)	8.7	4.0*	4.7	8.5	4.3*	4.3	0.447
	[7.8–9.6] ± 1.6	[3.1–4.9] ± 1.6	[3.6–5.9] ± 2.1	[7.5–9.5] ± 1.8	[3.2–5.3] ± 1.9	[3.6–5.0] ± 1.3	
GR (mm)	1.3	0.9	0.4	1.5	1.6	−0.1	0.809
	[0.9–1.7] ± 0.7	[0.3–1.5] ± 1.1	[−0.1–0.9] ±0.9	[0.8–2.1] ± 1.1	[0.3–2.8] ± 2.3	[−1.4–1.2] ±2.3	
Radiographic measurements^a^							
DD (mm)	5.9	0.7*	5.1	5.3	0.9*	4.4	0.245
	[5.2–6.6] ± 1.2	[0.4–1.1] ± 0.6	[4.4–5.9] ± 1.3	[4.3–6.3] ± 1.8	[0.6–1.2] ± 0.6	[3.4–5.4] ± 1.8	
RVG angle (degrees)	23.24			26.0			
	[21.4–25.5] ± 3.8	–	–	[22.2–29.9] ± 7.0	–	–	
Tooth type (*n*)							
Incisors	4	–	–	5	–	–	
Premolars	4	–	–	3	–	–	
Molars	7	–	–	7	–	–	
Intrasurgical measurements (mm)							
Defect depth	5.5	–	–	5.3	–	–	
	[4.7–6.4] ± 1.6			[4.1–6.6] ± 2.3			
Defect width	3.5	–	–	2.9	–	–	
	[2.6–4.4] ± 1.6			[2.5–3.3] ± 0.7			
Defect morphology							
One-wall	2	–	–	3	–	–	
Two-wall	4	–	–	4	–	–	
Three-wall	9	–	–	8	–	–	

*PPD* probing pocket depth, *CAL* clinical attachment level, *GR* gingival recession, *DD* defect depth, *n* number of defects, *p* intergroup comparison of the change

*Significantly different compared to baseline (*p* < 0.001)

^a^The means with 95% CI [in brackets] and ± SD of probing values and radiographic measurements of the defects

**Table 2:** Mean percentages of full-mouth plaque score (FMPS) and full-mouth bleeding score (FMBS)

	FMPS (%)	FMBS (%)
Baseline	8.45 [5.67–11.23] ± 7.46	12.61 [10.76–15.73] ± 7.3
1 month	12.66 [9.14–16.18] ± 9.42	12.41 [9.23–15.59] ± 8.51
3 months	14.32 [10.35–18.30] ± 10.66	14.41 [10.87–17.95] ± 9.48
6 months	16.65 [11.09–22.21] ± 13.77	14.60 [10.17–19.04] ± 10.98
12 months	19.88 [13.46–26.30] ± 17.20	19.75 [15.64–23.86] ± 11.01

The results show the means with 95% CI [in brackets] and ± SD

*FMPS* full-mouth plaque score, *FMBS* full-mouth bleeding score

**Fig. 2:**
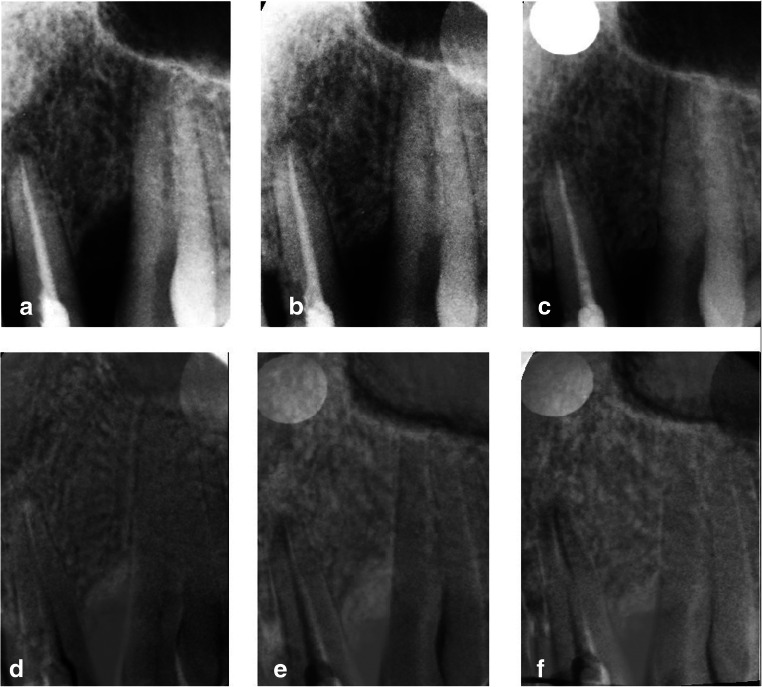
Subtraction radiography of test site (MPM-treated). Intrabony defect on mesial surface of tooth 23. **a**–**c** Radiographs after normalization. **a** Baseline radiograph. **b** Six-month radiograph. **c** Twelve-month radiograph. **d**–**f** Subtraction images. **d** Subtraction of 6-month radiograph from baseline radiograph. **e** Subtraction of 12-month radiograph from baseline radiograph. **f** Subtraction of 12-month radiograph from 6-month radiograph. Density gain as compare between 6 and 12 months post-operatively

**Fig. 3:**
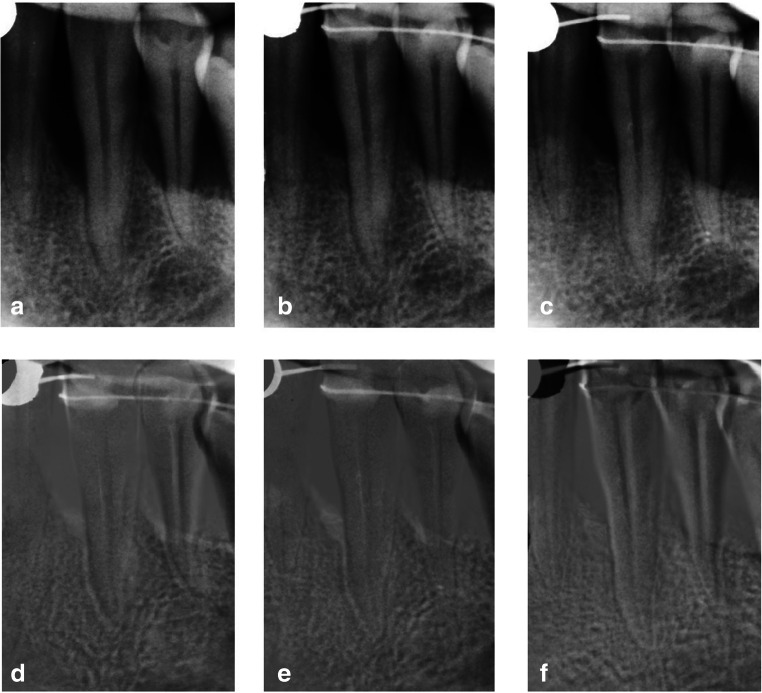
Subtraction radiography of test site (CM-treated). Intrabony defect on mesial surface of tooth 33. **a**–**c** Radiographs after normalization. **a** Baseline radiograph. **b** Six-month radiograph. **c** Twelve-month radiograph. **d**–**f** Subtraction images. **d** Subtraction of 6-month radiograph from baseline radiograph. **e** Subtraction of 12-month radiograph from baseline radiograph. **f** Subtraction of 12-month radiograph from 6-month radiograph. Minimal density gain as compare between 6 and 12 months post-operatively

When compared to the baseline, the outcomes of subtraction radiography at 6 and 12 months post-operatively showed no significant differences between the groups (Table 3). Both treatments demonstrated substantial gain in bone/graft density. However, a noticeable density gain at 12 months compared to bone/graft density at 6 months occurred to be significantly greater at the MPM sides (Figs. 1,2, and 3).

**Table 3:** Mean changes in bone density of the test and control sites at 6 and 12 months post-surgery

	Test (*n* = 15)	Control (*n* = 15)	*p* value
Mean change in bone density in subtraction at 6 months (compared to baseline)^a^	84.7% [0.79–0.91] ± 0.107	83.4% [0.76–0.91] ± 0.129	0.746
Mean change in bone density in subtraction at 12 months (compared to baseline)^b^	88.9% [0.85–0.93] ± 0.078	82.5% [0.74–0.91] ± 0.148	0.156
Mean change in bone density in subtraction at 12 months (compared to 6 months)^c^	4.9%^d,e^ [0.02–0.08] ± 0.058	− 0.8%^d^ [− 0.02 to 0.03] ± 0.04	0.011
*p* value (mean change in bone density between 12 months and 6 months within the same group)	0.008	0.585	

^a^The results show the means with 95% CI [in brackets] and ± SD of subtraction radiographic outcomes 6 months post-operatively compared to baseline

^b^The results show the means with 95% CI [in brackets] and ± SD of subtraction radiographic outcomes 12 months post-operatively compared to baseline

^c^The results show the means with 95% CI [in brackets] and ± SD of subtraction radiographic outcomes 12 months post-operatively compared to subtraction radiographic outcomes at 6 months

^d^Shows significant differences (*p* < 0.05) between values of the mean changes in subtraction radiographic outcomes between 12 and 6 months following surgery between test and control

^e^Shows significant differences (*p* < 0.05) between values of the mean changes in subtraction radiographic outcomes between 12 and 6 months following surgery within the same group

The analysis on the impact of defect type on the clinical outcomes showed significantly higher PPD reduction in the test group for type B defects (Table 4). Similar trend was apparent for CAL gain, and it almost reached statistical significance (*p* = 0.052). With regard to subtraction radiography outcomes, there was no significant difference detected between the type A and B defects for the MPM- and CM-treated sites.

**Table 4:** Comparison of the clinical and subtraction radiography outcomes according to defect type between test and control

	Test (*n* = 15)				Control (*n* = 15)				
	*n*	Baseline (*n* = 15)	12 months (*n* = 15)	Change (*n* = 15)	*n*	Baseline (*n* = 15)	12 months (*n* = 15)	Change (*n* = 15)	*p*
PPD^a^									
Type A	6	6.0 [5.06–6.94] ± 0.89	3.83 [3.04–4.62] ± 0.75	2.16 [0.77–3.56] ± 1.33	7	7.14 [5.79–8.50] ± 1.46	3.78 [2.60–4.98] ±	3.35 [2.08–4.63] ± 1.38	0.143
Type B	9	8.33 [7.47–9.19] ± 1.12	3.11 [2.13–4.09] ± 1.27	5.22 [4.02–6.42] ± 1.56	8	7.25 [6.28–8.22] ± 1.16	3.62 [3.19–4.06] ± 0.52	3.62 [2.63–4.62] ± 1.19	0.033*
CAL^a^									
Type A	6	7.33 [6.25–8.42] ± 1.03	4.0 [1.52–6.48] ± 2.37	3.33 [0.79–5.88] ± 2.42	7	8.42 [6.11–10.75] ± 2.51	4.28 [1.91–6.66] ± 2.56	4.14 [2.79–5.50] ± 1.46	0.473
Type B	9	9.66 [8.73–10.61] ± 1.22	4.0 [3.14–4.86] ± 1.12	5.66 [4.65–6.68] ± 1.32	8	8.62 [7.74–9.51] ± 1.06	4.25 [3.18–5.32] ± 1.28	4.37 [3.38–5.37] ± 1.19	0.052
Mean change in bone density^b^									
Type A	6	N/A	N/A	0.87 [0.78–0.96] ± 0.08	7	N/A	N/A	0.86 [0.78–0.93] ± 0.08	0.765
Type B	9	N/A	N/A	0.90 [0.84–0.96] ± 0.08	8	N/A	N/A	0.80 [0.64–0.96] ± 0.19	0.148

*n* number of defects, *PPD* probing pocket depth, *CAL* clinical attachment level, *type A* one-wall and two-wall defects, type B three-wall defects; *p* intergroup comparison of change

*Shows significant differences (*p* < 0.05) between the data at 12 months post-surgery and the values at baseline between test and control

^a^The means with 95% CI [in brackets] and ± SD of probing values at baseline and 12 months post-operatively

^b^The results show the means with 95% CI [in brackets] and ± SD of the changes in subtraction radiography outcomes 12 months post-operatively relative to baseline

At 12 months, PPD ≤ 4 mm was recorded in 12 defects (80%), CAL gain > 3 mm in 11 defects (73%), bone/graft density gain > 70% at 12 months post-operatively as compared to baseline in 15 defects (100%) and bone/graft density gain > 0 at 12 months as compared to 6 months in 11 defects (73%) in MPM sites. As for CM-treated sites, PPD ≤ 4 mm was recorded in 13 defects (87%), CAL gain > 3 mm in 11 defects (73%), bone/graft density gain > 70% at 12 months post-operatively as compared to baseline in 12 defects (80%) and bone/graft density gain > 0 at 12 months as compared to 6 months in 7 defects (47%). In stepwise multivariate analysis, no variable was significantly related to post-operative PPD. On the other hand, defect morphology was significantly related to CAL gain; the likelihood that post-operative CAL gain was > 3 mm was 8.09 times higher if treatment was performed in three-wall intrabony defects than in less contained defects. (Table 5). For changes in bone/graft density 12 months post-operatively compared to baseline, multivariate analysis indicated that radiographic angle was significantly associated parameter. Failure to achieve > 70% bone/graft density gain decreased for each 1-degree increase in radiographic angle. Radiographic defect depth was significantly related to alterations in bone/graft density from 6 to 12 months post-operatively assessed with radiographic subtraction. The probability of failure (no changes in bone/graft density or bone/graft density loss) was reduced by 2.33 times with each 1-mm gain in radiographic defect depth. Neither treatment modality (MPM/CM) nor type of healing (primary/secondary) significantly impacted outcomes.

**Table 5:** Multivariate models based on stepwise logistic regression

Model	Treatment outcome	Predictor	Category or Unit	OR	*p*
				[95% CI]	
Model I	CAL gain	Defect morphology	Number of walls	8.09	0.006
				[1.83–35.69]	
Model II	Change in bone density in subtraction at 12 months (compared to baseline)	Radiographic angle	1 degree	0.67	0.033
				[0.47–0.97]	
Model III	Change in bone density in subtraction at 12 months (compared to 6 months)	Radiographic defect depth	1 mm	2.33	0.025
				[1.11–4.90]	

*CAL* clinical attachment level

Table 6 presents the prevalence and extent of post-operative morbidity. The majority of patients reported some discomfort, pain, and edema with mild intensity without any significant differences between treatment modalities. One year after treatment, all patients were satisfied with the results of the surgery (MPM sites: VAS 92.13 ± 9.84; CM sites 92.93 ± 7.19, with 0 = no satisfaction and 100 = maximum satisfaction), and they would make the same decision regarding treatment if they had to (VAS 94.01 ± 6.84 and 93.86 ± 7.50, respectively).

**Table 6:** Subject experience in terms of post-operative morbidity (*N* = 15)

	Test (*n* = 15)		Control (*n* = 15			Test (*n* = 15)		Control (*n* = 15)		
	Number of subjects (%)	Mean ± SD	Number of subjects (%)	Mean ± SD	*p* value	Intensity (VAS) Mean ± SD	Minimum-maximum	Intensity (VAS) Mean ± SD	Minimum-maximum	*p* value
Discomfort	11 (73.3%)	0.73 ± 0.46	12 (80.0%)	0.80 ± 0.41	0.679	31.33 ± 27.56	0–74	30.47 ± 26.59	0–75	0.931
Pain	11 (73.3%)	0.73 ± 0.46	12 (80.0%)	0.80 ± 0.41	0.679	27.60 ± 24.01	0–72	30.40 ± 24.39	0–72	0.754
Intake of analgesics tablets	6 (40.0%)	1.00 ± 1.41	6 (40.0%)	1.27 ± 1.83	0.659					
Edema	10 (66.7%)	0.67 ± 0.49	11 (73.3%)	0.79 ± 0.43	0.491	24.93 ± 25.23	0–74	28.73 ± 22.70	0–57	0.668
Eating impairment	11 (73.3%)	0.79 ± 0.43	10 (66.7%)	0.67 ± 0.49	0.491	30.27 ± 24.48	0–71	33.13 ± 29.44	0–76	0.774
Speaking impairment	8 (53.3%)	0.53 ± 0.52	9 (60.0%)	0.60 ± 0.51	0.724	14.13 ± 19.21	0–57	18.20 ± 23.97	0–75	0.612
Interferences with daily activities	8 (53.3%)	0.47 ± 0.52	8 (53.3%)	0.53 ± 0.52	0.726	8.13 ± 11.40	0–32	11.60 ± 16.51	0–60	0.509
Interferences with work	7 (46.7%)	0.33 ± 0.49	7 (46.7%)	0.47 ± 0.52	0.473	4.33 ± 8.98	0–27	10.27 ± 16.09	0–50	0.223

*VAS* units, visual analogue scale units (0 = no discomfort and 100 = unbearable discomfort), *SD* standard deviation

## Discussion

In the present study, we evaluated GTR of intrabony defects treated with modified perforated (tests) or standard (controls) collagen membranes in patients diagnosed with AgP. Owing to their lack of rigidity, CMs are incapable of maintaining space for regeneration; hence, we used them in combination with DBBM as a filler. Both treatment modalities led to significant clinical (CAL gain, PPD reduction) and radiographic improvement (DD reduction) 12 months post-operatively, without significant intergroup differences. At MPM sites, the observed PPD reduction was 4.0 ± 2.1 mm, CAL gain 4.7 ± 2.1 mm, and DD reduction 5.1 ± 1.3 mm (the respective values for CM sites were 3.5 ± 1.2 mm, 4.3 ± 1.3 mm, 4.4 ± 1.8 mm). However, when analyzing the impact of remaining walls of intrabony defects on the clinical and radiographic outcomes, we observed significantly higher PPD reduction for three-wall defects in the MPM sites, when compared to CM sites. PPD reduction in the test group was 5.22 ± 1.56 mm, in contrast to 3.62 ± 1.19 mm in the control group. Similar positive trend was apparent for CAL gain, as greater CAL gain was noted in the test group (MPM sites 5.66 ± 1.32; CM 4.37 ± 1.19; *p* = 0.052). Even though all defect types were included in this study, they were classified in subgroups as type A (one-wall and two-wall) and type B (three-wall) for separate analysis due to the small sample size. The findings of this study highlight the clinical relevance of MPM for the treatment of intrabony defects in AgP patients. These results might suggest the beneficial input of MPM to the healing of more contained intrabony defects. MPM had a dense collar that might inhibit epithelial downgrowth on the outer surface of the graft and perforated body that could allow MSCs from periosteum and gingival connective tissue to contribute to the regenerative processes. Moreover, it has been reported that growth and differentiation factors could pass through membrane perforations and promote regeneration processes [29]. Analysis of the gingival crevicular fluid in sites treated with MPM showed elevated concentration of bone morphogenetic protein-2 (BMP-2), vascular endothelial growth factor (VEGF), and platelet-derived growth factor-BB (PDGF-BB), which enhanced the clinical outcomes of periodontal regeneration [29, 30]. Membrane perforations might also play an important role in stabilizing the formed fibrin clot in intrabony defects due to mechanical interlocking of fibrin strands with the pores on the one side, together with fibrin clot integration with gingival connective tissue on the opposing side [14].

Another aim of this report was to measure the bone/graft density gain in intrabony defects by means of DSR following GTR treatment. In subtraction radiographs taken 6 and 12 months post-operatively, the areas representing mean gain in density were 84.7 and 88.9% of the baseline defect for MPM sites and respectively 83.4 and 82.5% for CM sites, with no significant differences between the groups. However, there was a statistically significant density gain of 4.9% in the test sites, from 6 to 12 months after GTR, which may indicate greater area of new bone formation (*p* = 0.008). The present results reflect the value of MPM for the regenerative treatment of intrabony defects in AgP patients. The significantly higher density gain that was observed at the MPM sites could also reflect the enhanced osteogenic effect of periosteal active beneficence through membrane perforations, as explained previously. Though DSR grants visualization of changes between two images, it is also dependent on standardization of the radiographic images taken at different times. In the present study, the radiographs were standardized by means of applying the parallel technique with individual film holders and customized bite indices, and computer analysis. It should be underlined that the resorption rate of DBBM is really slow, and in cases of using this material in combination with GTR, the observed defect fill will amount to a blend of both radiopaque xenogenic graft particles and regenerating vital human bone. As a consequence, the results of DSR may not represent a true bone formation/maturation because of the residual particles of bone grafts. Accordingly, a healing period with longer span seems to be more accurate for precise radiographic evidence of true bone formation to become discernible [31–33]. In agreement with the results of the present study, Rakmanee et al. [31] observed that the bone fill and defect resolution were higher at 12 months than at 6 months after GTR in AgP indicating that bone regeneration might be still an ongoing process at 6 months after the surgery.

The results of this study match the conclusions of most recent systematic review of the existing literature, that GTR could be successfully implemented in patients affected by AgP [34]. However, only three papers were published on two RCTs, which tested different biomaterials and surgical techniques [31, 32, 35]. Rakmanee et al. [35] reported PPD reduction of 2.4 mm and CAL gain of 1.6 mm at the GTR sites with CM without fillers and PPD reduction of 2.5 mm and CAL gain of 2.1 mm at the access flap sites 12 months post-operatively. No statistically significant differences could be demonstrated between groups and both treatment showed significant improvement in radiographic bone fill (2.2 and 2.6 mm, respectively) [31, 35]. The authors suggested that PPD reduction and CAL gain following GTR might partly be influenced by the morphology of the initial defects, and the baseline defect angle could be a predictor of the potential CAL gain. Queiroz et al. [32] compared treatment outcomes of intrabony defects with anorganic bone matrix/cell binding peptide (ABM/P15) or GTR with titanium reinforced non-absorbable ePTFE membrane at 6 months. At the GTR sites, authors found PPD reduction of 2.57 mm, CAL gain of 2.09 mm, and bone fill of 0.73 mm, as compared to PPD reduction of 2.27 mm, CAL gain of 1.87, and bone fill of 2.49 mm at the GTR sites (*p* > 0.005). In subtraction radiographs, the areas representing gain in density were 62.03% of the baseline defect for GTR group and 93.16% in ABM/P-15 group (*p* = 0.011). All things considered, none of the biomaterials were found to be more advantageous than others, while the small number of RCTs restricted the chance of performing a quantitative analysis of the results. This is in part because of widely heterogenous study populations and varying subject numbers. The present findings compare well to those from the abovementioned clinical trials.

The general treatment goals do not remarkably differ for AgP and ChP. However, the extensive amount of bone loss in respect to the young age of patients with pathological migration of teeth and early tooth mobility might affect the psychological well-being of patients, which is why a comprehensive and predictable treatment plan is required for AgP cases. A retrospective study spanning 6.6 years showed that patients with AgP have a significantly faster linear pattern of progression than do patients with ChP: 0.31 versus 0.20 mm/year, respectively [36]. Owing to the low prevalence of AgP and difficulties in the recruitment of sufficient numbers of subjects for RCTs, the response to GTR in those patients is much less recorded and understood, as pinpointed in the previous paragraph. The evidence available on the GTR outcomes has been granted mainly by uncontrolled studies and short follow-ups. It seems from the literature that AgP is more challenging to treat than ChP [13]. On the other hand, the clinical response to GTR in the treatment of ChP has been well documented. A meta-analysis by Laurell et al. [37] reported that GTR resulted in significant PPD reduction from 8.5 to 3.4 mm, CAL gain of 4.2 mm, and bone fill averaging 3.2 mm. CAL gain and bone fill correlated significantly (*p* < 0.001) with defect depth (*R* = 0.52 and 0.53 respectively), and to benefit from GTR treatment, the depth of intrabony defect has to be at least 4 mm. A recent study utilized inflammatory serum markers as treatment outcomes observed that despite comprehensive periodontal therapy and significant clinical improvement, neutrophil elastase (NE) and C-reactive protein (CRP) levels in patients with AgP were significantly elevated compared to patients with ChP even 5 years after periodontal treatment [38]. When comparing responses to GTR treatment, it should be underscored that compliance with supportive periodontal therapy among patients with AgP and ChP may differ. Significantly better compliance was reported among patients with AgP (57.7%) than among those with ChP (30.6%) [39]. As AgP results in rapid destruction of alveolar bone, tooth mobility, pathological tooth migration, and early tooth loss, fear of such consequences could improve compliance.

Within the scope of periodontal regenerative procedures, treatment outcomes are influenced by various patient-, and technique-associated factors. Although the importance of these variables is well established in ChP, sparse data exists with respect to their impact on AgP treatment. The influence of patient-related variables may be substantially governed by cause-related therapy and by individual selection of the suitable case. As a proof of principle, it has been established that suboptimal plaque control, smoking, and non-participation in a regular oral hygiene protocol exert a negative impact on short- and long-term results following GTR therapy [25, 40–42]. It must be emphasized that the outcomes of periodontal regenerative treatment are highly dependent on scrupulous patient selection. In the reported study, due to strict inclusion criteria and post-operative protocol, patient-related variables were rigorously controlled. Moreover, flap design, surgical execution, and strict maintenance regimen were accurately dealt with; hence, the present study assessed only site-related factors. To the best of our knowledge, no studies have determined so far the potential preoperative factors associated with improved outcomes after GTR of intrabony defects in AgP patients in multivariate analysis. In an attempt to do so, we decided on explicit endpoints, such as post-operative PPD ≤ 4 mm, CAL gain > 3 mm, and gain in bone/graft density > 70%, since PPD ≤ 4 mm has been correlated with greater long-term stability, whereas abovementioned CAL gain and defect resolution are regarded as favorable outcomes [43]. Consequently, multivariate analysis identified three prognostic factors that independently impacted on treatment outcomes.

No parameter was significantly related to post-operative PPD in multivariate analysis. Of note, in the study by Parashis et al. [44], the probability of post-operative PPD > 4 mm increased 1.6 times with each 1-mm baseline PPD increase in intrabony defects treated with enamel matrix derivative (EMD) in ChP. Though deep pockets have a higher potential for improvement, these pockets will still represent unfavorable post-treatment parameters as residual pockets are associated with substantial loss of CAL and increases in GR [45]. Nevertheless, we identified significant independent predictor of positive treatment outcomes in terms of CAL gain. Regression analysis showed that the probability of gaining ≥ 3 mm CAL was 8.09 times higher if treatment was performed in three-wall defects than in less contained defects. The reason for which it may be attributed to the adequate stability of the blood clot and its protection in the long term that could be procured in cases of self-containing intrabony defects. Thus, the morphology of the intrabony defect might be a crucial determinant influencing post-operative outcomes [12, 44]. Several authors have demonstrated a similar association in ChP [33, 40, 46–48]. Although the present findings are difficult to compare directly to those obtained in ChP patients, the majority of reports points clearly to the pivotal role of intrabony defect morphology to obtain predictable periodontal regeneration. Larger distances required for cellular repopulation of the wound entails higher chance of incomplete bone fill [49]. What is more, greater amounts of CAL and bone can be gained in deeper intrabony defects [33, 35, 40]. It has been demonstrated that intrabony defects deeper than 3 mm were associated with greater CAL gain than defects of 3 mm or less [46]. Moreover, the depth of three-wall intrabony components determined CAL gain [50]. In the present study, the defects had an average baseline PPD of 7.3 mm [6.5–8.3], baseline CAL of 8.6 mm [7.5–9.6], and baseline defect depth of 5.4 mm [4.1–6.6]. These parameters were relatively higher than characteristics in the study of Rakmanee et al. [35], which may account for bigger PPD reduction and greater CAL gain in our research.

Likewise, radiographic defect depth and angle were significantly associated with alterations in bone/graft density evaluated by DSR. The probability of achieving > 70% density gain at 12 months as compared to baseline declined as the angulation score decreased, whereas the chance of additional gain in bone/graft density at 12 months as compared to 6 months increased as the DD deepened. Cortellini and Tonetti [47] classified intrabony defects with RVG angle ≤ 25% as narrow and ≥ 37% as wide and reported a 1.5-mm greater defect fill in narrow defects. Rakmanee et al. [31] reported more significant bone fill in narrow (≤ 19°) and deep defects than in wide (≥ 31°) and shallow defects in AgP. In different study on ChP, bony fill could be predicted by baseline depth and angulation of bony defect [41]. In narrow (< 37°) and deep (≥ 4 mm) infrabony defects, bony fill was more prominent than in wide and shallow defects. The different cut-off values between all the studies were based on interquartile ranges of the data distribution. By any means, wider defects might be more prone to environmental influences and to the collapse of degradable barrier membranes, due to their lack of rigidity. However, these findings have not been universally demonstrable. One multicenter study has accounted no relationship between presurgical defect angle and clinical outcomes after GTR with DBBM and CM with a papilla preservation flap in ChP patients [51]. These results are in agreement with our observations, and they may pinpoint to a substantial influence of the additional use of bone fillers, which may reduce the negative impact of unfavorable intrabony defect morphology. With this in mind, the importance of intrabony defect characteristics with respect to its depth, width, and angle, which might hinder the measurements of bone/graft density gain analyzed by subtraction radiography, should be accentuated. Similarly, in narrow defects, the bone filling may result in a small density gain, while in wide defects, larger bone filling contributes to substantial gain in bone/graft density. However, the density gain may not necessarily be the evidence of true regeneration, as some authors observed no correlation between subtraction radiography outcomes and CAL gain [52].

Taking everything into consideration, understanding how well the treatment modality in question works, and the predictability to obtain significant CAL gain and shallow pockets are of paramount importance to the clinical decision making. Respectively, our research presents support for the regenerative management of intrabony defects in AgP patients and pinpoints some variables with prognostic value. These data may be of great assistance to the clinician in choosing regenerative strategy of intrabony defects in patients with AgP in terms of safety and predictability, based on the presurgical factors. In the present study, we found that the association between treatment modality (MPM/CM) and outcomes did not reach statistical significance in multivariate analysis, and thus concluded that the type of collagen membrane used in GTR is not independent factor influencing 12-month clinical or radiographic treatment outcomes. However, the absence of evidence is not automatically evidence of absence; hence, identifying independent prognostic variables with multivariate analysis represents a major challenge. It should be understood that once several characteristics are included in analysis, multicollinearity could happen. This is especially important when there are high correlations among predictor variables, as for multivariate analysis to give more confident results, it needs a large sample of data. That is to say, some prognostic variables, such as treatment modality, could have been identified in the present study if the sample was larger.

It has been urged to underline the effects of regenerative approaches on different aspects of patients’ quality of life, as well as treatment satisfaction, so as to better understand the areas of concern to patients [53]. However, there is a paucity of research including patient-reported outcomes when analyzing regenerative approaches [53]. It is of utmost importance to decipher post-operative morbidity and patient perceptions of long-term satisfaction with treatment, especially in the light of considering alternative treatment scenarios. To assess how much GTR affect the different aspects of patient life, the following components should be taken into account: (1) level of discomfort/pain, (2) functioning, (3) psychological, and (4) social well-being [53]. All of the abovementioned issues were evaluated in the reported study. The majority of patients experienced discomfort (tests: VAS 31.22 ± 27.56; controls: VAS 30.47 ± 26.59 with 0 = no discomfort and 100 = unbearable discomfort) and pain (tests VAS 27.60 ± 24.01; controls 30.40 ± 24.39 with 0 = no pain and 100 = unbearable pain) of mild intensity. Ten patients did not take any analgesic in addition to the first two compulsory tablets that were administrated after the surgery. Post-surgical, slight edema was observed at 66% (10) test sites and 73% (11) control sites. Seventy-three percent (11) of patients after GTR with MPM and 66% (10) patients after GTR with CM mentioned eating impairment, while the respective values for problems with speaking were 53% (8) and 60% (9). Fifty-three percent (8) of patients reported some interferences with daily activities while 46% (7) little interferences with work. Overall, there were no significant differences when it comes to early post-operative morbidity between surgical treatment with MPM or CM. Although flap reflection was kept to a minimum, in order to advance flaps coronally without tension to fully cover barrier membrane, periosteal incisions were performed in all cases. Flaps were passively sealed with modified internal mattress sutures. Despite complete gingival wound closure after surgery, the membrane exposed in 5 sites (16.7%). All of the abovementioned factors might account for patient early morbidity. It should be emphasized though that neither membrane exposure nor reported discomfort during early post-operative period affected clinical or subtraction radiography outcomes. However, small number of sites with membrane exposure may have mitigated the effect of this variable in the study. Twelve months after surgery, all patients reported very high level of satisfaction with the treatment outcomes. Generally speaking, after GTR with resorbable barrier membranes, significantly more edema may be expected with regard to access flap alone [54]. However, barrier membrane exposure with subsequent bacterial contamination epitomizes the major complication of GTR with collagen membranes, with prevalence in the range of 20–68% [55]. Reports in the literature on the effect of membrane exposure on the long-term treatment outcomes are conflicting. A meta-analysis that evaluated the effect of membrane exposure on the obtained clinical outcomes showed that the sites with exposed membranes had a significantly reduced CAL gain (4.22 mm) than the sites without membrane exposure (4.69 mm) (*p* < 0.05) [56]. On the other hand, the Cochrane review by Needleman [55] reported on the modest effect of the membrane exposure on healing, although such an event could necessitate more rigorous maintenance or the use of systemic antibiotics. In contrast, very limited morbidity was associated with minimally invasive surgical technique with an enamel matrix derivative [26, 57]. The short surgical time, limited surgical trauma, and the stability of the flaps could possibly account for the limited post-operative patient morbidity. While incorporating patient-reported outcomes in periodontal clinical trials, the type of questions being asked and the timing of those questions are important research design concerns [58].

In interpreting these data, one should understand that only histological examination would be able to determine the true range of periodontal regeneration, but it was not possible due to ethical concerns. That is why in the present study, changes in probing measurements and subtraction radiography evaluation were used to assess effects of GTR. Keeping in mind that DBBM is radiopaque and very slowly reabsorbed, it is unattainable to distinguish by radiographic methods whether it was replaced by vital bone. Other limitation includes the restrictions in the sample size that may impinge on the fact that some potential variables with prognostic values might have been overlooked due to reduced power of statistical analysis. Multicollinearity should also be considered when evaluating a multivariate analysis results. Thus, the results of subgroup analyses should be interpreted cautiously. However, other RCTs used sample sizes similar to those of the present research. The present findings highlight that identification of prognostic parameters of GTR outcomes in AgP patients before treatment may have a plausible clinical application. Moreover, reevaluation of periodontal status at 6 weeks after non-surgical therapy is debatable due to clinical and radiographic improvements carrying on for at least 6 to 9 months [59]. All things considered, more RCTs correlating the characteristics of the intrabony defects, alterations in bone density (or bone/graft density), and improvements in probing parameters would prompt the assessment of regenerative treatment of intrabony defects in AgP patients. By the same token, future well-designed clinical trials with longer follow-up and larger sample size are required to further evaluate and confirm the efficacy of MPM for periodontal regeneration and the long-term stability of clinical outcomes.

## Conclusions

In conclusion, and within the limitations of this study, it seems that:

1. Both GTR with MPM or CM resulted in significant gain in CAL, reduction in PPD, and reduction in radiographic DD at 12 months. However, the use of MPM led to significantly higher PPD reduction for three-wall defects that increased the efficiency of periodontal treatment.
2. Both treatment strategies resulted in significant bone/graft density gain evaluated with subtraction radiography at 6 and 12 months, without significant intergroup differences. Nevertheless, there was a statistically significant density gain of 4.9% in the test sites from 6 to 12 months after GTR; thus, the modification of collagen membranes may have positive impact on the new bone formation, which can yet be the continuous process 6 months after treatment.
3. Intrabony defect morphology (the number of remaining bony walls) might be a predictor of CAL gain, while radiographic baseline defect depth and angle might represent predictors of changes in bone/graft density after regenerative treatment of intrabony defects in AgP patients.
4. After GTR with either MPM or CM in AgP post-operative discomfort, pain and edema of mild intensity, as well as eating impairment, might be expected without significant differences between treatment modalities. Still, long-term patient-satisfaction with treatment outcomes can be very high.
